# Opioids Impair Intestinal Epithelial Repair in HIV-Infected Humanized Mice

**DOI:** 10.3389/fimmu.2019.02999

**Published:** 2020-01-17

**Authors:** Jingjing Meng, Santanu Banerjee, Li Zhang, Greg Sindberg, Shamsudheen Moidunny, Bin Li, David J. Robbins, Mohit Girotra, Bradley Segura, Sundaram Ramakrishnan, Sabita Roy

**Affiliations:** ^1^Department of Surgery, University of Miami, Miami, FL, United States; ^2^Department of Surgery, University of Minnesota, Minneapolis, MN, United States; ^3^Department of Pharmacology, University of Minnesota, Minneapolis, MN, United States; ^4^Department of Veterinary Biosciences, University of Minnesota, Saint Paul, MN, United States; ^5^Division of Gastroenterology, University of Miami, Miami, FL, United States

**Keywords:** opioids, HIV, gut microbiota, BLT mice, intestinal organoid, notch

## Abstract

Intestinal barrier dysfunction and subsequent microbial translocation play crucial roles in persistent immune activation leading to HIV disease progression. Opioid use is associated with worse outcome in HIV-infected patients. The exacerbated disease progression by opioids is mainly driven by excessive intestinal inflammation and increased gut permeability. The objective of this study is to investigate how opioids potentiate HIV disease progression by compromising intestinal barrier function and impairing intestinal epithelial self-repair mechanism. In the present study, abnormal intestinal morphology and reduced epithelial proliferation were observed in bone marrow-liver-thymus humanized mice and in HIV-infected patients who were exposed to opioids. In bone marrow-liver-thymus mice, HIV, and morphine independently, and additively induced gut dysbiosis, especially depletion of Lachnospiraceae, Ruminococcaceae, and Muribaculaceae. We also observed that the abundance of Lachnospiraceae, Ruminococcaceae, and Muribaculaceae negatively correlated with apoptosis of epithelial cells, and intestinal IL-6 levels. Previous studies have shown that these bacterial families play crucial roles in maintaining intestinal homeostasis because they include most short-chain fatty acid-producing members. Short-chain fatty acids have been shown to maintain stem cell populations and suppress inflammation in the gut by inhibiting histone deacetylases (HDAC). In addition, we demonstrate that morphine exposure inhibited growth of intestinal organoids derived from HIV transgenic mice by suppressing Notch signaling in an HDAC-dependent manner. These studies implicate an important role for HDAC in intestinal homeostasis and supports HDAC modulation as a therapeutic intervention in improving care of HIV patients, especially in opioid-abusing population.

## Introduction

Opioid users are a substantial fraction of the total HIV-infected population in the United States. Opioids are prescribed for pain management in HIV-infected patients at rates higher than that in the general population, with some studies suggesting that more than 50% of the people with HIV were actively being treated with opioids (1, 2). In addition to prescription use for pain management, illicit opioid use was found in 37% of HIV+ patients in a study performed at seven primary care sites in the United States (3). Despite the high incidence of opioid use/abuse in HIV patients, very few studies have investigated the effects of opioids on disease progression and outcome of HIV infection. A recent collaborative analysis of cohort studies showed that although the mortality of HIV patients continued to decline during the first 3 years of anti-retroviral therapy, the decline in mortality was less in people who inject drugs, the population who frequently used opioids (4). However, the mechanistic role of HIV-opioid interaction in disease progression still remains poorly understood.

Recent studies indicate that compromised intestinal barrier function and subsequent microbial translocation contribute to persistent immune activation and chronic inflammation in HIV-infected patients. This ongoing immune activation is linked to CD4^+^ T-cell depletion and disease progression (5). Moreover, growing evidence shows that gut microbial dysbiosis contributes to abnormal tissue inflammation and gut leakage following opioid exposure (6). Furthermore, studies demonstrate that the gut microbiome in opioid users was distinct from healthy controls (7). Similarly, a corroborating study demonstrated that in patients with cirrhosis, chronic morphine exposure reduced the levels of beneficial short-chain fatty acid (SCFA)-producing bacteria in the patients' microbiome, correlating with endotoxemia and up-regulation of pro-inflammatory cytokine IL-6 (8).

Until now, however, there has been no mechanistic study investigating how opioids promote HIV disease progression by disrupting gut homeostasis. To better understand the interplay between gut immunity, HIV, and opioids, BLT (bone marrow, liver, thymus) humanized mice (9) were infected with HIV and treated with morphine. The intestinal sections from BLT mice and patients were used to determine whether this small animal HIV-infection model could mirror what is seen in humans. In addition, intestinal organoid cultures were established to explore the mechanisms by which HIV and opioids disrupt gut homeostasis. The gut sections from HIV-infected and morphine-treated mice displayed disrupted epithelial barriers and decreased epithelial proliferation, which was consistent with the observations in patients. We also demonstrated that gut microbial dysbiosis induced by morphine and HIV was associated with abnormal histone deacetylase (HDAC) activities and impaired intestinal self-renewal.

## Methods

### Generation of Humanized Mice

Humanized BLT mice were generated by implanting xenogenic thymus and liver tissue into 4 week-old NOD-scid IL2R gamma null mice (The Jackson Laboratory) as previously described (10). Briefly, implants consisted of minced fetal liver tissue and fetal thymus. Each implant was put into the right kidney capsule. In addition to the implants, the mice also received CD34+ hematopoietic stem cells isolated from fetal liver using CD34 Microbead Kit (Miltenyi Biotec). All humanized mice were maintained in a specific pathogen-free facility according to protocols approved by University of Minnesota (UMN) Institutional Animal Care and Use Committee. Humanization levels were determined in these mice 13–15 weeks after CD34+ cell injection. For that, peripheral blood was obtained from animals via submandibular venipuncture and collected into EDTA tubes. Whole peripheral leukocytes were stained for anti-human and anti-mouse CD45 antibodies and analyzed with flow cytometry. All animals with more than 25% human CD45^+^ cells among the combined CD45 population were used for further experimentation.

### HIV Infection and Morphine Treatment

Patient-derived HIV isolates (ADA-M) were obtained from National Institutes of Health AIDS repository and expanded on human monocytes (donor-derived; Red Cross of America) for 14 days, with media and monocyte replenishment every 48–72 h. At the end, media were harvested and centrifuged at 500 g, and HIV concentration was measured using HIV p24 antigen ELISA kit (ZeptoMetrix Corporation, Buffalo, NY). Based on the concentration, appropriate aliquots were made and stored in −80°C until further use.

Appropriate groups of fully characterized BLT humanized mice were injected with HIV particles (10 ng of calculated p24 antigen/mice) for 4 weeks. In the combined HIV and morphine treatment groups, morphine was administered after 21 days of HIV infection. A small incision was made at the dorsal torso of the mice. The placebo or 75 mg slow release morphine pellet was inserted into the small pocket created during the incision, and the wound was closed using stainless steel wound-clips. The BLT mice were sacrificed 7 days after morphine treatment.

### Histological Evaluation

Hematoxylin and eosin (H&E) staining was performed by Comparative Pathology Shared Resource at UMN. The pathological score was evaluated using histological scoring system as previously described (11). To assess the damage induced by intestinal inflammation, the villi were scored (ranges from 0 to 12 points) on the basis of epithelial morphology, villus shape, retracted stroma, infiltration, and crypt shape.

### Immunohistochemistry (IHC) and Immunofluorescence

IHC image of activated notch in formalin fixed, paraffin embedded intestinal tissue sections was obtained on an IHC Select® HRP/DAB system (Millipore) according to the manufacturer's protocol. The section was incubated with anti-activated Notch1 antibody (Abcam) for 16 h at 4°C. For immunofluorescence images, formalin-fixed, paraffin-embedded sections were processed and incubated with anti-Ki67 antibody (Abcam), anti-claudin-1 antibody (Invitrogen™), and anti-zona occuldens (ZO)-1 antibody (Invitrogen™). The terminal deoxynucleotidyl transferase-mediated deoxyuridine triphosphate nick end-labeling (TUNEL) assay was performed using Click-iT® Plus TUNEL assay kit (Invitrogen™) following the manufacture's protocol.

### Gut Microbiome Analysis

The fecal content was collected from the cecal-colonic region of mice upon necropsy. The gut tissues were fixed and processed separately. The fecal matter was lysed using glass beads in MagnaLyser tissue disruptor (Roche), and the total DNA was isolated using a Power-soil/fecal DNA isolation kit (Qiagen) as per the manufacturer's specifications. All samples were quantified via the Qubit® Quant-iT dsDNA Broad-Range Kit (Invitrogen, Life Technologies), to ensure that they met the minimum concentration and mass for DNA and submitted to UMN Genome Center for microbiome analysis as follows. To enrich the sample for the bacterial 16S V4 rDNA region, DNA was amplified using fusion primers designed against the surrounding conserved regions, which are tailed with sequences to incorporate Illumina (San Diego, CA) flow cell adapters and indexing barcodes. Each sample was PCR amplified with two differently bar-coded V4 fusion primers and was submitted for pooling and sequencing. For each sample, amplified products were concentrated using a solid-phase reversible immobilization method for the purification of PCR products and quantified by electrophoresis using an Agilent 2100 Bioanalyzer®. The pooled 16S V4-enriched, -amplified, -barcoded samples were loaded into the MiSeq® reagent cartridge and then onto the instrument along with the flow cell. After cluster formation on the MiSeq instrument, the amplicons were sequenced for 250 cycles with custom primers designed for paired-end sequencing. Using QIIME2 and custom scripts, the sequences were quality filtered and demultiplexed using exact matches to the supplied DNA barcodes. The resulting sequences were then searched against the Silva (release 132) reference database for 16S sequences and clustered at 99% by uclust [closed-reference Operation Taxonomic Unit (OTU) picking] (12). The longest sequence formed from each OTU was then used as the OTU representative sequence and assigned a taxonomic classification.

### Crypt Isolation and Organoid Culture

Intestinal organoid cultures were established from WT and NL4-3 Δ *gag/pol* transgenic (Tg26) mice, as previously described (13). Briefly, the small intestine was opened longitudinally and washed with cold phosphate-buffered saline (PBS) to remove luminal content. The tissue was then cut into 2 to 4 mm pieces and further washed 10 times with cold PBS by pipetting up and down using a 10 ml pipette. Tissue fragments were incubated with Gentle Cell Dissociation Reagent (STEMCELL Technologies) for 15 min at room temperature. After removal of the Cell Dissociation Reagent, tissue fragments were washed with PBS to release crypts. Supernatant fractions enriched in crypts were collected, passed through a 70 μm cell strainer, and centrifuged at 300 g for 5 min. The cell pellet was resuspended with Dulbecco's modified Eagle medium/F12 medium and centrifuged at 200 g. Tg26 mouse expresses a 7.4-kb transgene that contains the genetic sequence for all HIV-1 proteins except gag and pol (14). This mouse line expresses HIV-specific RNA in various tissues including the gastrointestinal tract. Tg26 mice in the C57BL/6 background were obtained from Dr. Roy Lee Sutliff's laboratory (Emory University School of Medicine, Atlanta, GA) and were maintained as heterozygous lines in accordance with the National Institutes of Health guidelines and regulations of the Institutional Animal Care and Use Committee of UMN and the University of Miami. Crypts were then entrapped in Matrigel (growth factor reduced; BD Bioscience) and cultured using advanced Dulbecco's modified Eagle medium/F12 containing various growth factors in the presence or absence of 1 μM morphine.

### Human Samples

Human tissues were obtained from the National Disease Resource Interchange as well as Bionet histology resources of UMN. The information of patients is listed in Supplementary Table 1. Representative intestinal H&E images for patients are shown (Supplementary Figure 3). The institutional review board of UMN determined that this project does not meet the regulatory definition of human subject research, and hence, no further institutional review board review/approval was required.

### Statistics

For microbiome analysis, QIIME 2 was used to calculate the α diversity and to summarize taxa. Principal coordinate analysis was used to visualize inter-object similarity/dissimilarity in a low-dimensional, Euclidean space. The test of significance was PERMANOVA with 999 permutations, generating false discovery rate-adjusted *P*-value (*Q*-value). *Q* < 0.05 was considered to be statistically significant. For other experiments, ANOVA followed by Bonferroni correction was used (GraphPad Prism). *P* < 0.05 was considered to be statistically significant. All the *P* or *Q*-values for each experiment were indicated in the figure legends.

## Results

### Morphine Treatment Exacerbated Intestinal Epithelial Damage in HIV-Infected BLT Mice

**Figure 1:**
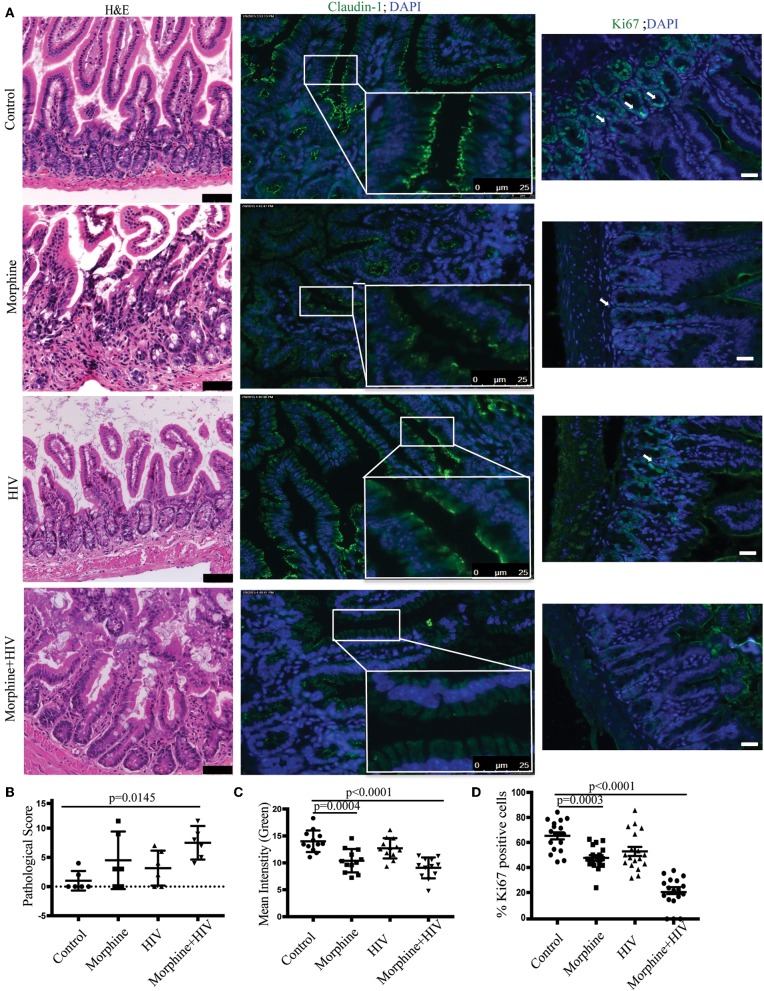
Morphine treatment potentiated intestinal epithelial damage in HIV-infected BLT (bone marrow, liver, thymus) mice. **(A)** Small intestinal sections harvested from the healthy controls, morphine-treated mice, the HIV-infected mice, and HIV-infected morphine-treated mice were stained with hematoxylin and eosin (H&E) (left anel; scale bar: 50 μm). Immunofluorescence was performed for claudin-1 (middle panel) and Ki67 (right panel; scale bar: 25 μm; white arrow: Ki67^+^ cells). **(B)** Intestinal tissue damages were scored based on a histological scoring system. Each dot represents one animal (*n* = 6). ANOVA followed by Bonferroni correction, *F*_(3, 20)_ = 4.001, ANOVA *P* = 0.0221. *P*-values for group comparison are shown in the figure if smaller than 0.05. **(C)** Claudin-1 (green) expression levels were quantified by measuring the intensity of green fluorescence within each villus. Quantification from two different fields for each animal is depicted (*n* = 12). ANOVA followed by Bonferroni correction, *F*_(3, 44)_ = 14.79, ANOVA *P* < 0.0001. *P*-values for group comparison are shown in the figure if smaller than 0.05. **(D)** The percentage of Ki-67^+^ (green) cells in each crypt was quantified. Quantification from three different crypts for each animal is depicted (*n* = 18). ANOVA followed by Bonferroni correction, *F*_(3,68)_ = 43, ANOVA *P* < 0.0001. *P*-values for group comparison are shown in the figure if smaller than 0.05.

To explore the mechanisms by which opioids and HIV disrupt gut homeostasis, we generated the BLT mice as an animal model of HIV infection. HIV infection in BLT mice was validated using p24 Elisa and HLA-DR+CD4+ cell counts. Reduced number of HLA-DR+CD4+ cells in blood and increased p24 expression in plasma indicated active HIV infection 4 weeks after intravenous injection of HIV (Supplementary Figure 1). H&E sections were used to investigate the effects of opioids and HIV infection on morphology of intestines. Histological analysis indicated abnormal morphology of the small intestinal villi in the HIV-infected and morphine-treated mice compared with healthy controls (Figure 1A). Significant loss of epithelial continuity and increased immune cell infiltration were observed in HIV/morphine mice. Histological scores indicated the most severe tissue injury in HIV/morphine mice (Figure 1B). Analysis of tight junction (TJ) proteins by immunofluorescence microscopy indicated that both opioids and HIV infection induced disrupted barrier integrity in the small intestines of patients (Figure 1A). A significant reduction in the expression of trans-membrane TJ protein Claudin-1 was observed in vehicle/morphine and HIV/morphine mice (Figures 1A,C). Although HIV or opioids did not change ZO-1 expression in BLT mice, we still observed disorganization of ZO-1 in HIV/morphine animals (Supplementary Figure 2). These data suggest an impairment of TJ organization and a dysfunction of the epithelial barrier in HIV-infected opioid abusers. Stem cell regeneration in intestinal crypts is an important self-repair mechanism that helps host maintain homeostasis in context of infection or tissue injury. To study the proliferation of crypt stem cells during HIV infection, the intestinal sections were stained with anti-Ki67 antibody. Regardless of the HIV-infection status, opioid-treated animals had significantly lower levers of epithelial proliferation (represented as the proportions of Ki67+ cells in each crypt), suggesting that opioids induced intestinal epithelial disruption by impairing crypt regeneration (Figures 1A,D).

Similar intestinal epithelial damage was observed in the HIV-infected patients taking opioids (Supplementary Figures 3A,B). Loss of epithelial cells and crypts were observed in HIV/opioid patients (Supplementary Figure 3A). A significant down-regulation of trans-membrane TJ Claudin-1 and para-cellular TJ ZO-1 was also observed in HIV/opioid patients (Supplementary Figures 3A,C, 4). Opioid exposure also resulted in a reduction in Ki-67 proliferative cell in crypts (Supplementary Figures 3A,D). These data were consistent with the results obtained from BLT humanized mice (Figure 1), indicating that the impaired intestinal homeostasis observed in BLT mice reflected response to HIV infection and opioid exposure in patients.

**Figure 2:**
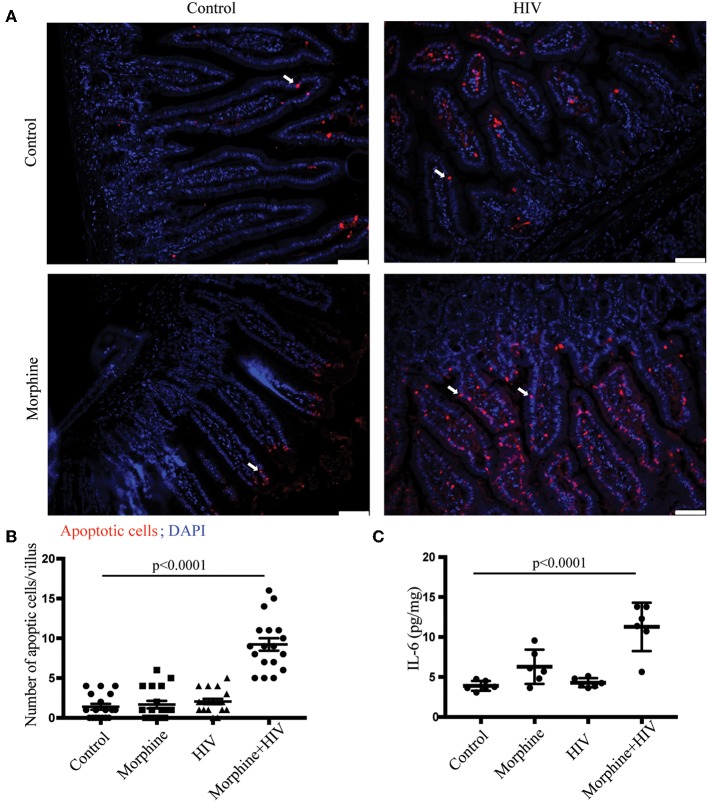
Morphine treatment induced mucosal cell apoptosis and tissue inflammation in the intestine of HIV-infected BLT mice. **(A)** Terminal deoxynucleotidyl transferase–mediated deoxyuridine triphosphate nick end-labeling (TUNEL) assay was used to detect apoptotic cells in the intestinal sections. Apoptotic cells were stained red. Scale bar: 50 μm; white arrow: apoptotic cells. **(B)** Quantification of the TUNEL assay. The number of apoptotic cells in each villus was quantified. Quantification from three different villi for each animal is depicted (*n* = 18). ANOVA followed by Bonferroni correction, *F*_(3,68)_ = 52.32, ANOVA *P* < 0.0001. *P*-values for group comparison are shown in the figure if smaller than 0.05. **(C)** ELISA was used to determine the expression levels of IL-6 in the intestine. Each dot represents one animal (*n* = 6). ANOVA followed by Bonferroni correction, *F*_(3, 20)_ = 19.13, ANOVA *P* < 0.0001. *P*-values for group comparison are shown in the figure if smaller than 0.05.

In addition, TUNEL assay was used to detect apoptotic cells in the intestine. The combination of morphine and HIV induced the greatest level of apoptosis (represented as the number of apoptotic cells per villus) in the intestinal epithelium (Figures 2A,B). ELISA assay using intestinal tissue showed that the pro-inflammatory IL-6 was significantly up-regulated in the small intestines of HIV/morphine mice (Figure 2C).

Using humanized mice, we showed that a combination of morphine treatment and HIV infection induced TJ disruption, impaired crypt regeneration, increased epithelial cell apoptosis, and increased pro-inflammatory cytokine expression in small intestines.

### Opioids Suppressed Notch Signaling in Intestinal Crypts in the Context of HIV Infection

**Figure 3:**
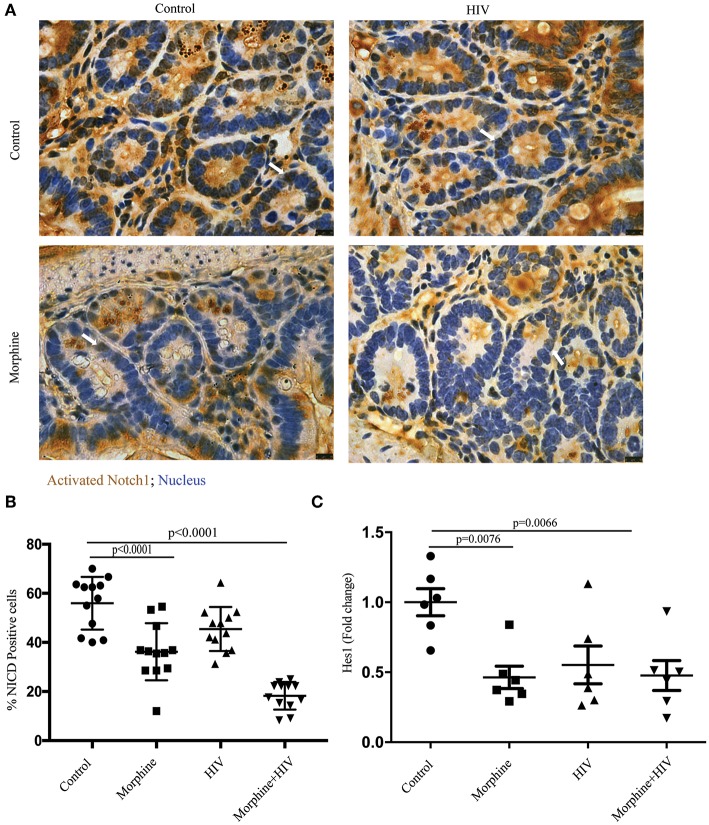
Morphine treatment suppressed Notch signaling pathway in the intestine of HIV-infected BLT mice. **(A)** Immunohistochemistry (IHC) was performed to determine activated Notch1 in the intestinal section. The positive cells were stained brown. Scale bar: 10 μm. **(B)** Quantification of IHC images. The number of positive cells in each crypt was quantified. Quantification from two different crypts for each animal is depicted (*n* = 12). ANOVA followed by Bonferroni correction, *F*_(3, 44)_ = 33.86, ANOVA *P* < 0.0001. *P*-values for group comparison are shown in the figure if smaller than 0.05. **(C)** PCR was used to determine the expression level of hairy and enhancer of split-1 (HES1) in the intestine. Each dot represents one animal (*n* = 6). ANOVA followed by Bonferroni correction, *F*_(3, 20)_ = 5.589, ANOVA *P* = 0.0060. *P*-values for group comparison are shown in the figure if smaller than 0.05.

Notch signaling is one of the primary signaling pathways that regulates apoptosis and regeneration of intestinal epithelial cells (15). To study the effects of opioid exposure and HIV infection on Notch signaling in the crypt cells, the intestinal sections were stained with anti-cleaved Notch1 antibody. Consistent with the reduced Ki67+ cells in crypts, we observed decreased Notch1 immunoreactivity in the crypts of opioid-treated individuals (Figures 3A,B). The results of real-time PCR also showed that the Notch signaling downstream target, HES1, was down-regulated by morphine in BLT mice (Figure 3C). This result indicates that morphine treatment reduces the activities of NOTCH signaling in intestinal crypts.

### Morphine Treatment and HIV Infection Induced Gut Microbial Dysbiosis

**Figure 4:**
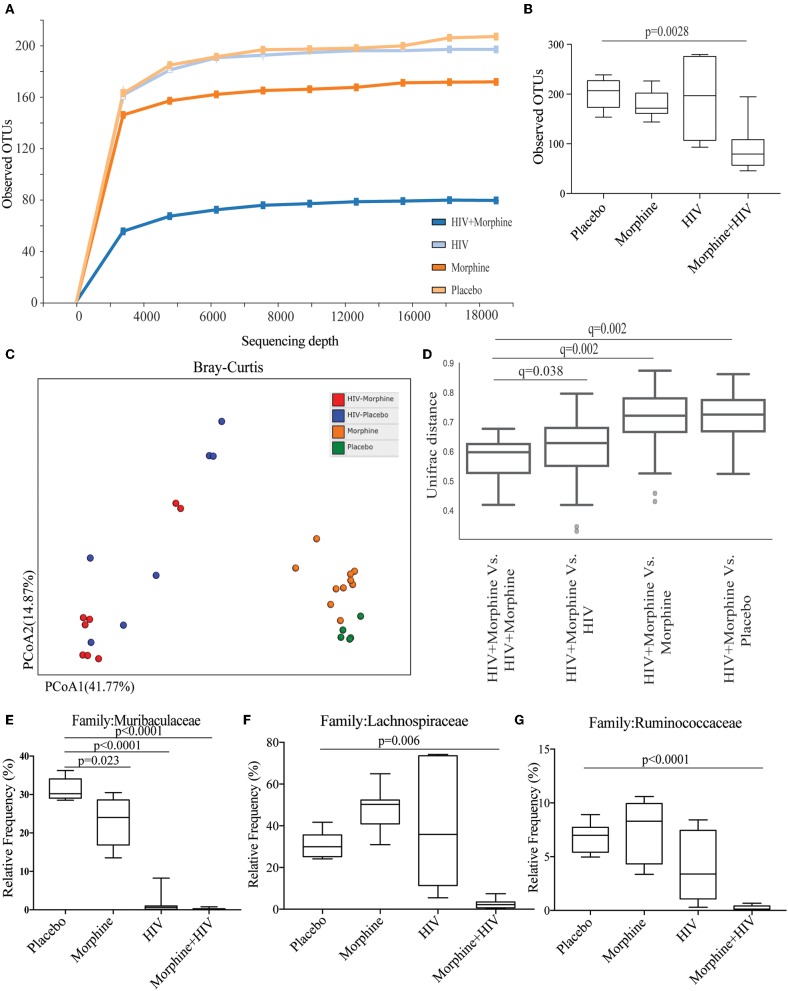
Morphine treatment and HIV infection induced gut microbial dysbiosis. **(A)** Alpha diversity from BLT mice infected with vehicle or HIV and treated with placebo or morphine was assessed by species richness [presented as observed Operation Taxonomic Units (OTUs)]. **(B)** Quantification of observed OTUs. ANOVA followed by Bonferroni correction, *F*_(3, 28)_ = 8.086, ANOVA *P* = 0.0005. *P*-values for group comparison are shown in the figure if smaller than 0.05. **(C)** Principal coordinate analysis (PCoA) of samples using the Bray_Curtis metrics at the OTU level shows distinct clustering of all four groups. **(D)** HIV + morphine group showed significant distance from all other groups. The test of significance was PERMANOVA with 999 permutations, generating false discovery rate-adjusted *P*-value (*Q*-value) **(E)** Relative abundance of Muribaculaceae. ANOVA followed by Bonferroni correction, *F*_(3, 28)_ = 107, ANOVA *P* < 0.0001. *P*-values for group comparison are shown in the figure. **(F)** Relative abundance of Lachnospiraceae. ANOVA followed by Bonferroni correction, *F*_(3, 28)_ = 16.48, ANOVA *P* < 0.0001. *P*-values for group comparison are shown in the figure if smaller than 0.05. **(G)** Relative abundance of Ruminococcaceae. ANOVA followed by Bonferroni correction, *F*_(3, 28)_ = 18.47, ANOVA *P* < 0.0001. *P*-values for group comparison are shown in the figure if smaller than 0.05.

To study the role of gut microbiome in morphine-induced intestinal inflammation, intestinal contents were collected for gut microbiome analysis based on Illumina sequencing of microbial 16S-rRNA genes. Sequencing data showed that the combination of morphine and HIV resulted in a significant reduction in alpha-diversity (presented as Observed OTUs and Shannon index) (Figures 4A,B and Supplementary Figure 5). Principal coordinate analysis showed that the microbiota from the morphine-treated groups clustered distinctly from the placebo groups (Figures 4C,D). In non-infected groups, the two dominant phyla were Firmicutes and Bacteroidetes. The significantly greater relative abundance of Firmicutes and Proteobacteria was observed in the HIV/morphine group (Supplementary Figures 5A–C). A significant decrease in the relative abundance of Bacteroidetes, Actinobacteria, and Tenericutes was also observed in HIV/morphine group (Supplementary Figures 6D–F). Furthermore, taxonomic analysis demonstrated that the combination of morphine and HIV resulted in a significant decrease in the relative abundance of Muribaculaceae, Lachnospiraceae, and Ruminococcaceae (Figures 4E–G). Previous studies have associated SCFA production in the gut with enrichment in Muribaculaceae, Lachnospiraceae, and Ruminococcaceae. These bacterial families play crucial roles in intestinal homeostasis because SCFAs display potent anti-inflammatory properties via HDAC inhibition (16–18). Consistent with these studies, we also observed a negative correlation of these bacterial communities with the number of apoptotic epithelial cells and intestinal IL-6 levels in our BLT mice (Supplementary Figure 7).

In addition, genus-level analysis indicated that HIV infection significantly increased the relative abundance of *Staphylococcus*, and the combination of HIV and morphine significantly increased the relative abundance of *Enterococcus* (Supplementary Figures 8B,C), which was consistent with our previous studies (19). Morphine treatment and HIV infection also reduced the relative abundance of beneficial bacteria *Lactobacillus* (Supplementary Figure 8D). The results of microbiome analysis showed that both morphine treatment and HIV infection gut microbial dysbiosis and the combination of morphine treatment and HIV infection specifically deplete beneficial bacteria such as SCFA-producing bacteria.

### Morphine Inhibited the Growth of Small Intestinal Crypts of Tg26 Mice

To understand the mechanism by which morphine modulates the proliferation of crypt stem cells, we established intestinal organoid cultures from WT and Tg26 mice following treatment with saline or morphine. The Tg26 transgenic mouse is a murine model for HIV infection, which express seven of the nine HIV-1 viral proteins under the viral long terminal repeat promoter.

**Figure 5:**
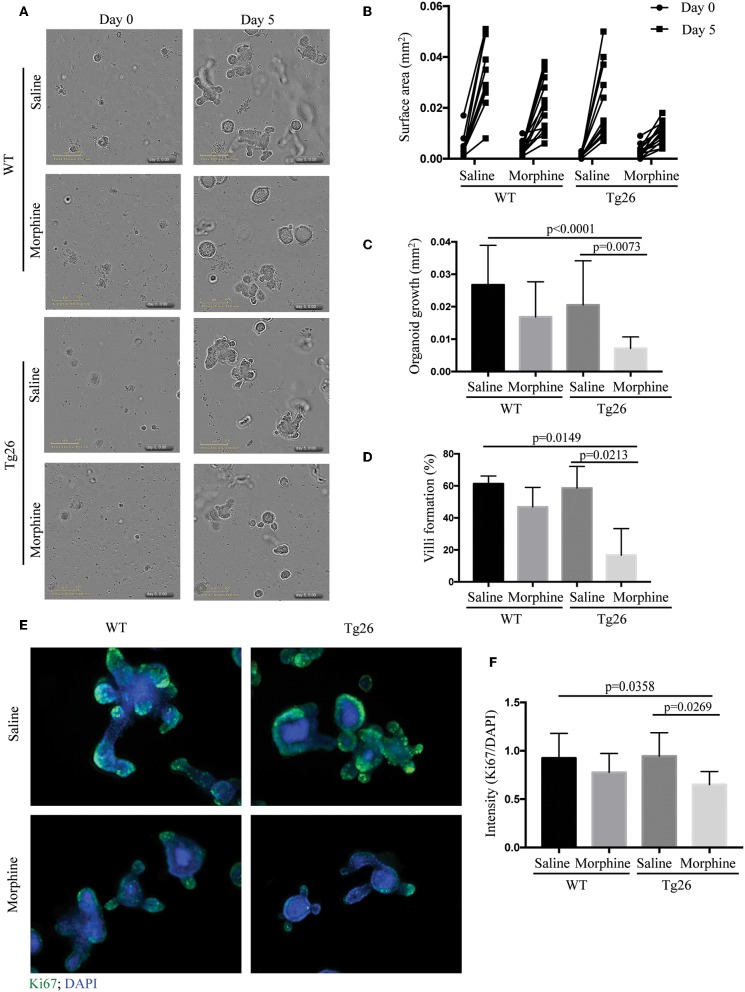
Morphine inhibited the growth of small intestinal organoids of Tg26 mice. **(A)** Saline- or morphine- treated WT or Tg26 intestinal organoids were isolated and cultured in the IncuCyte for live-imaging analysis for 5 days. The representative image of organoid culture is depicted. **(B)** The surface areas of each organoid on day 0 and 5 were quantified using ImageJ. Each dot represents one organoid (*n* = 15). **(C)** The increase of surface area was calculated by subtracting the surface area of day 0 from that of day 5 (*n* = 15). ANOVA followed by Bonferroni correction, *F*_(3, 56)_ = 8.711, ANOVA *P* < 0.0001. *P*-values for group comparison are shown in the figure if smaller than 0.05. **(D)** The percentage of organoids that can form villi (*n* = 3). ANOVA followed by Bonferroni correction, *F*_(3, 8)_ = 7.898, ANOVA *P* = 0.0089. *P*-values for group comparison are shown in the figure if smaller than 0.05. **(E)** Representative images of organoids stained with anti-Ki-67 antibody. **(F)** Quantification of Ki-67-stained organoids. DAPI (40,6-diamidino-2-phenylindole) was used as a nuclear stain. Green fluorescence intensity was normalized by DAPI (*n* = 10). ANOVA followed by Bonferroni correction, *F*_(3, 36)_ = 4.197, ANOVA *P* = 0.0120. *P*-values for group comparison are shown in the figure if smaller than 0.05.

The crypt organoids were cultured in the IncuCyte for live-imaging analysis for 5 days (Supplementary Videos 1–4 and Figure 5A). The results showed that morphine exposure significantly inhibited the growth of Tg26 organoids (Figures 5B,C). In addition, reduced villi formation was also observed in morphine-treated Tg26 organoids (Figure 5D). To further validate the effects of morphine, Ki-67 staining was used to label the proliferative cells in the organoids. Consistent with the IncuCyte data, fewer Ki-67-positive cells were detected in the morphine-treated Tg26 organoids (Figures 5E,F and Supplementary Figure 9). Consistent with our observation in humanized mice, morphine inhibits growth of intestinal organoids derived from Tg26 mice, further validating that morphine exposure impaired crypt regeneration in the context of HIV infection.

### Morphine Inhibited Notch Signaling in the Small Intestinal Crypts of Tg26 Mice

**Figure 6:**
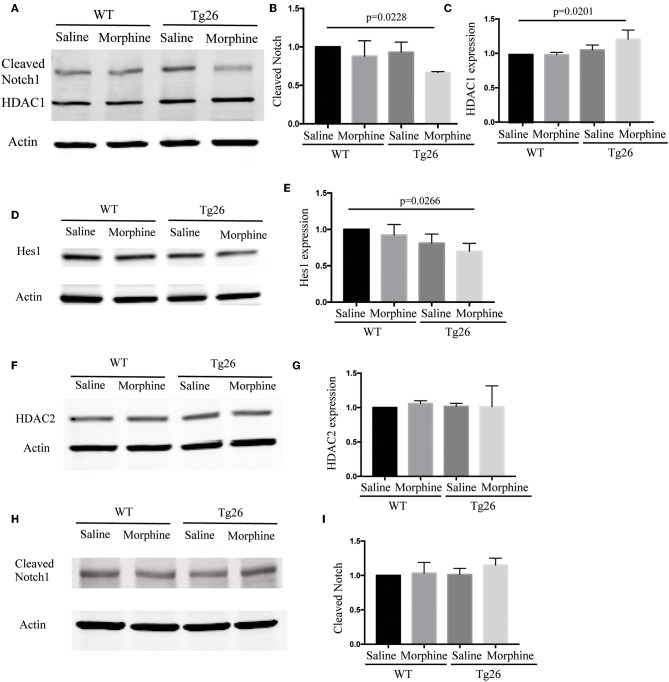
Morphine modulated Notch signaling in the intestinal crypts of Tg26 mice. **(A)** Representative Western blot analysis for cleaved Notch1 and HDAC1 in organoids. **(B)** Densitometric analysis of cleaved Notch1 (*n* = 3). ANOVA followed by Bonferroni correction, *F*_(3, 8)_ = 4.343, ANOVA *P* = 0.0429. *P*-values for group comparison are shown in the figure if smaller than 0.05. **(C)** Densitometric analysis of HDAC1 (*n* = 3). ANOVA followed by Bonferroni correction, *F*_(3, 8)_ = 5.562, ANOVA *P* = 0.0233. *P*-values for group comparison are shown in the figure if smaller than 0.05. **(D)** Representative Western blot analysis for HES1 in organoids. **(E)** Densitometric analysis of HES1 (*n* = 3). ANOVA followed by Bonferroni correction, *F*_(3, 8)_ = 4.171, ANOVA *P* = 0.0472. *P*-values for group comparison are shown in the figure if smaller than 0.05. **(F)** Representative Western blot analysis for HDAC2 in organoids. **(G)** Densitometric analysis of HDAC2 (*n* = 3). ANOVA followed by Bonferroni correction, *F*_(3, 8)_ = 0.0812, *P* = 0.9684. **(H)** Representative Western blot analysis for cleavd-Notch1 following HDAC inhibitor treatment. **(I)** Densitometric analysis of cleaved-Notch1 (*n* = 3). ANOVA followed by Bonferroni correction, *F*_(3, 8)_ = 1.348, *P* = 0.3258. Values were normalized against β-actin and presented as a ratio, compared with the control group. Data represent the means of three independent blots.

The effects of morphine on notch signaling in the intestinal organoids were investigated by determining the expression of cleaved Notch 1 and its downstream target HES1. Morphine treatment significantly suppressed the expression of cleaved Notch 1 and HES1 in Tg26 mice (Figures 6A–E), which was consistent with the observation in BLT mice.

HDAC and Notch signaling have been shown to interact with each other and play important roles in regulating stem cell maintenance, proliferation, and differentiation. In Tg26 mice, we showed that the HDAC inhibition efficiency of the intestinal contents was significantly down-regulated by morphine treatment (Supplementary Figure 10). Moreover, both up-regulation of HDAC1 and down-regulation of cleaved Notch1 were observed simultaneously in the morphine-treated Tg26 crypt organoids (Figures 6A–C), while HDAC2 was not affected by morphine treatment (Figures 6F,G). HDAC inhibitors have been shown to promote intestinal epithelial regeneration under inflammatory conditions (20). This was consistent with our observation that HDAC inhibitor suberoylanilide hydroxamic acid blocked the effects of morphine on Notch signaling in the organoids (Figures 6H,I), suggesting that morphine suppresses Notch signaling in Tg26 intestinal organoids in an HDAC-dependent manner. The whole images for the western blots are shown in Supplementary Figure 11. These studies implicate important roles of HDAC and NOTCH signaling in intestinal stem cell proliferation.

## Disscussion

Accumulating pieces of clinical evidence show that persistent immune activation and abnormally high inflammation following HIV infection are associated with accelerated disease progression to AIDS and death (21, 22). These observations lead to the hypothesis that progression to AIDS following HIV infection is driven by chronic immune activation and systemic inflammation. The etiology of persistent immune activation is attributed to impaired gut mucosal immune function and subsequent microbial translocation across the compromised gut epithelial barrier. However, the current literature cannot explain the poorer disease outcomes in HIV-infected patients who frequently use opioids.

To better understand the mechanisms by which opioids exacerbate HIV disease progression by disrupting gut homeostasis, HIV-infected-BLT mice were used to investigate intestinal pathophysiology during HIV infection with and without morphine treatment. We observed significant changes in the bacterial composition of morphine-treated and HIV-infected animals. Gut microbiome of HIV-infected animals showed enriched Proteobacteria and depletion of Bacteroidetes, which was consistent with previous clinical studies (5, 23). At the family level, morphine potentiated enrichment of Enterobacteriaceae in BLT mice, supporting the clinical observation that Enterobacteriaceae positively correlated with microbial translocation and inflammatory markers (IL-1β and IFN-γ) (24). We also observed depletion of Muribaculaceae, Lachnospiraceae, and Ruminococcaceae. These bacterial families include most of SCFA producers and play important roles in maintaining gut homeostasis, since SCFAs are crucial mediators that regulate gut immune responses and maintain intestinal epithelial integrity (16–18). In addition, SCFAs can be absorbed from the gut lumen to the periphery and modulate the functions of multiple organs or cells including liver, immune cells, and even CNS21. SCFAs, principally butyrate, are the preferred energy substrates for normal colonocytes (25). Reduced capacity for butyrate production by the gut microbiome is observed in patients with active Crohn's disease or ulcerative colitis (25, 26). Furthermore, butyrate also enhances the barrier function of intestinal epithelial cells by up-regulating TJ protein claudin-1 (27). As HDAC inhibitors, butyrate, and valproic acid are important for maintaining stem cell populations, and stimulating normal cell growth in small intestinal organoid culture (13, 28). SCFAs also induce different effects on immune cells in a cell- and environment-specific context. Via HDAC inhibition, SCFAs can suppress pro-inflammatory cytokine production by lamina propria macrophages and monocyte-derived dendritic cells (29), block differentiation of dendritic cells from bone marrow stem cells (29), and facilitate extrathymic generation of regulatory T cells (30). Overall, consistent with the previous studies, our study provides more evidence suggesting that depletion of SCFA-producing bacteria in intestines is one of the important contributors to intestinal epithelial disruption and tissue inflammation during in HIV-infected opioid abusers.

At the genus level, *Staphylococcus* and *Enterococcus* were enriched in HIV-infected BLT mice treated with morphine. Both are known opportunistic pathogens and sources of bacteremia in HIV-infected subjects (31) and patients on opioids (32). We also observed a significant decrease in *Lactobacillus* in morphine-treated and HIV-infected animals. *Lactobacillus* is an essential and common inhabitant of the human intestine, and various members of *Lactobacillus* exert anti-apoptotic, and anti-inflammatory effects (33). Taken together, the results from BLT mice demonstrate that morphine treatment induced potentiation of gut microbial dysbiosis. Future studies are needed to better understand the outcomes of the depletion of beneficial bacteria and enrichment of pathogenic bacteria in gut microbiota, which will be crucial for us to predict the outcome of disease progression in HIV patients.

Opioids have been shown to inhibit tissue regeneration and repair in different diseases. Barlass et al. report that morphine exacerbated the severity of acute pancreatitis by inhibiting regenerative response of pancreas and promoting tissue inflammation in the pancreas (34). Prescription opioids were associated with reduced likelihood of chronic wound healing (35), and further animal studies demonstrated that opioids prevent tissue regeneration through inhibition of ROS production (36). Eisch et al. also reported that morphine influenced hippocampal function by inhibiting neurogenesis in the adult rat hippocampus (37). In our study, lower epithelial Ki67 was observed in both opioid abusers and morphine-treated BLT mice, indicating that opioids also influenced tissue regeneration in intestinal crypts. A previous study had shown that crypt epithelium express both μ and σ opioid receptors, and these receptors contribute to opioid-mediated regulation of intestinal secretion (38). However, the effects of opioids on crypt stem cell growth still remain unknown. To further delineate the mechanisms of how opioid and HIV infection modulate the growth of intestinal crypt cells, we isolated saline- or morphine-treated WT and Tg-26 intestinal crypts and established crypt organoid culture, and tracked their growth using the IncuCyte system. Morphine significantly inhibited the growth of Tg26 organoids and down-regulated activated Notch1 in intestinal crypts. These data support the important role of Notch signaling in intestinal epithelial homeostasis and crypt regeneration. Previous studies have shown that deletion of Notch1 resulted in reduced expression of Olfm4 and fewer LGR5^+^ stem cells in intestinal crypts and caused impaired crypt regeneration after radiation in mice (15). The suppressive effects of opioids on Notch signaling have been observed in the brain of mice (39, 40), and our study confirmed that opioid exposure exerts similar effects on the Notch pathway in intestinal crypts.

In the morphine-treated Tg26 organoid culture, down-regulation of cleaved Notch1 and up-regulation of HDAC1 were observed, suggesting a link between HDAC and Notch signaling. The effects of morphine on cleaved Notch were abolished when treated with HCAC inhibitor, suberoylanilide hydroxamic acid, demonstrating that morphine's effects on Notch were mediated by HDAC. In recent years, HDACs have become a focus of much research given their important roles in growth and differentiation of various cells, including stem cells and immune cells. For example, HDAC1 has been shown to regulate retinal neurogenesis in zebrafish by suppressing Notch and Wnt signaling (41). Both epithelial HDAC1 and HDAC2 contribute to regulation of the intestinal inflammatory response by regulating intestinal epithelial cell proliferation and differentiation (42). The HDAC inhibitor valproic acid is commonly used with GSK-3 inhibitor Chiron99021 in early passages for organoid culture to improve the efficiency of organoid formation because they can up-regulate Wnt and Notch signaling, resulting in the presence of more Lgr5+ stem cells (13, 28). Interestingly, Langlands et al. found that the combination of valproic acid and Chiron99021 restricts the growth advantage of Apc mutant (mutations in Adenomatous polyposis coli) organoid cells while stimulating the growth of wild-type cells, implying that HDAC inhibitors selectively protect the normal crypt stem cells in the intestine (28). HDAC inhibition also attenuated morphine tolerance in a bone cancer pain model and facilitated extinction of morphine-induced conditioned place preference, suggesting that the interactions of HDAC and opioid receptors were crucial for host responses to opioid exposure (43).

On the other hand, HDACs are modulated by multiple factors. In addition to SCFAs produced by gut microbiota as described above, both HIV and morphine have been shown to modulate HDAC expression. Up-regulation of HDAC2 in neurons by HIV TAT was associated with down-regulation of CREB and CaMKIIa genes that are known to regulate neuronal activity, suggesting the possible role of HDAC in HIV-associated neurocognitive disorder (44). Previous studies by Tsai et al. showed that morphine induced HDAC1 up-regulation in rat spinal cord dorsal horn, which contributed to neuro-inflammation and subsequent tolerance (45). Therefore, it will be interesting to investigate the detailed mechanisms by which HIV and opioids modulate HDAC activities, and future studies will also examine how abnormal HDAC activities regulate host response to opioids and HIV.

Despite the remarkable success of ART, some vulnerable populations, especially opioid abusers, do not fully benefit. Our work explores the mechanisms by which opioids exacerbate HIV disease progression by disrupting gut homeostasis using patient intestinal samples and BLT mice. The results demonstrate that opioid-induced potentiation of gut microbial dysbiosis is associated with compromised intestinal barrier function, impaired epithelial regeneration, and exacerbated intestinal inflammation in HIV-infected individuals. Better understanding of these mechanisms will facilitate the further development of novel diagnostic tools and therapeutic interventions to improve patient care for HIV-infected patients, especially in the opioid-abusing population. Our study reveals a novel and important role of gut microbiota-induced HDAC inhibition in intestinal regeneration and inflammation and supports the application of probiotics as a useful adjunct therapy to control intestinal inflammation in HIV patients.

## Data Availability Statement

The microbiome datasets supporting the conclusions of this article is available in the ArrayExpress repository. The accession number is E-MTAB-7883.

## Ethics Statement

Ethical review and approval was not required for the study on human participants in accordance with the local legislation and institutional requirements. Written informed consent for participation was not required for this study in accordance with the national legislation and the institutional requirements. The animal study was reviewed and approved by IACUC at University of Minnesota and University of Miami.

## Author Contributions

JM, SB, and SRo: conception and design. JM, SB, LZ, GS, BL, DR, SM, and BS: acquisition of data. JM, SB, LZ, GS, BL, DR, SM, BS, MG, SRa, and SRo: writing, review, and revision of the manuscript.

### Conflict of Interest

The authors declare that the research was conducted in the absence of any commercial or financial relationships that could be construed as a potential conflict of interest.
