# Supplementary material for: Opioids Impair Intestinal Epithelial Repair in HIV-Infected Humanized Mice
Source: Front Immunol. 2020 Jan 17;10:2999. doi: 10.3389/fimmu.2019.02999 (PMC6978907; doi:10.3389/fimmu.2019.02999)
Supplement: Supplementary file 5 [file Presentation_1.PPTX]

## Slide 1
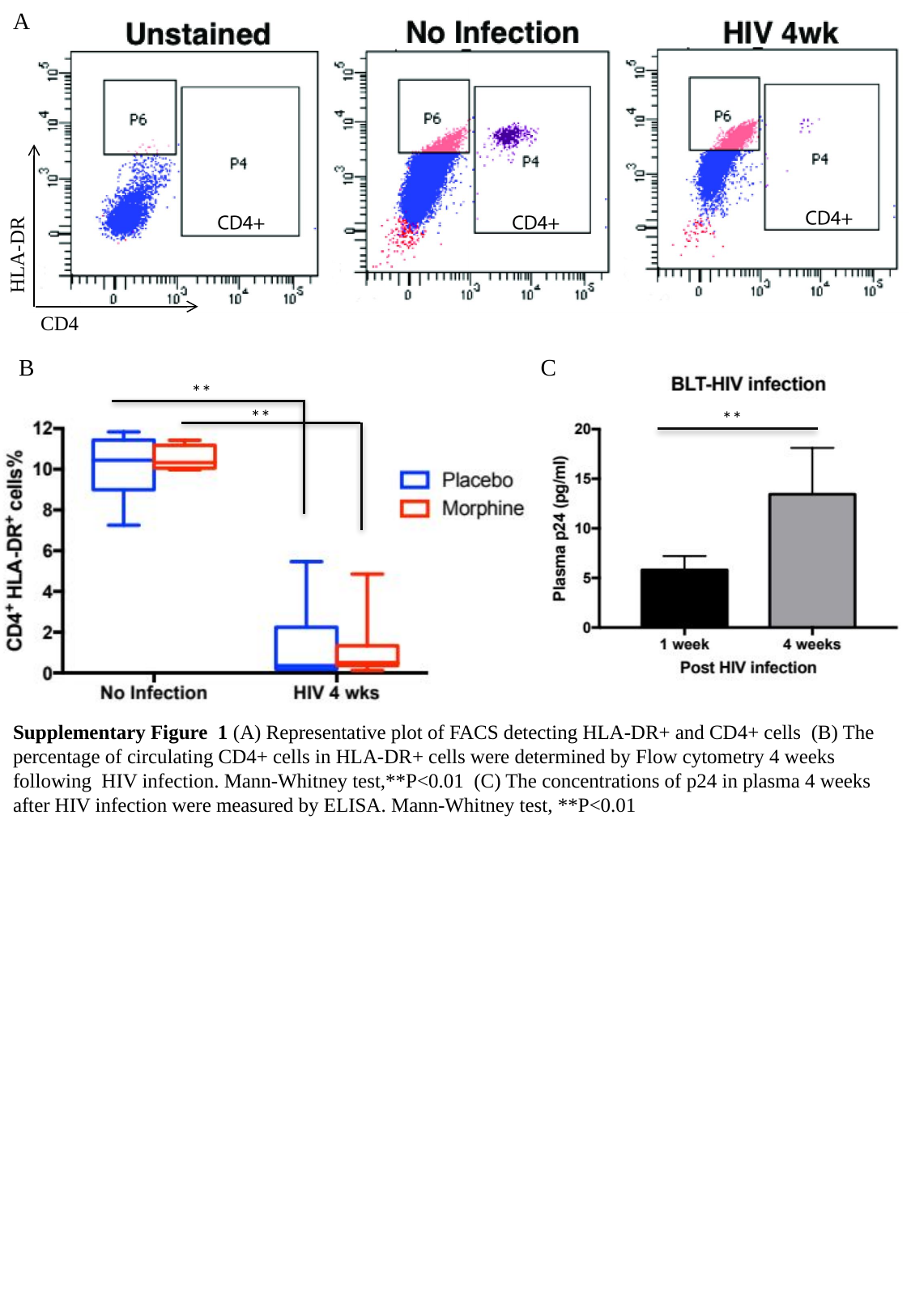

A
HLA-DR
CD4
B
C
**
**
**
Supplementary Figure 1 (A) Representative plot of FACS detecting HLA-DR+ and CD4+ cells (B) The percentage of circulating CD4+ cells in HLA-DR+ cells were determined by Flow cytometry 4 weeks following HIV infection. Mann-Whitney test,**P<0.01 (C) The concentrations of p24 in plasma 4 weeks after HIV infection were measured by ELISA. Mann-Whitney test, **P<0.01
