# Supplementary material for: Opioids Impair Intestinal Epithelial Repair in HIV-Infected Humanized Mice
Source: Front Immunol. 2020 Jan 17;10:2999. doi: 10.3389/fimmu.2019.02999 (PMC6978907; doi:10.3389/fimmu.2019.02999)
Supplement: Supplementary file 6 [file Presentation_2.PPTX]

## Slide 1
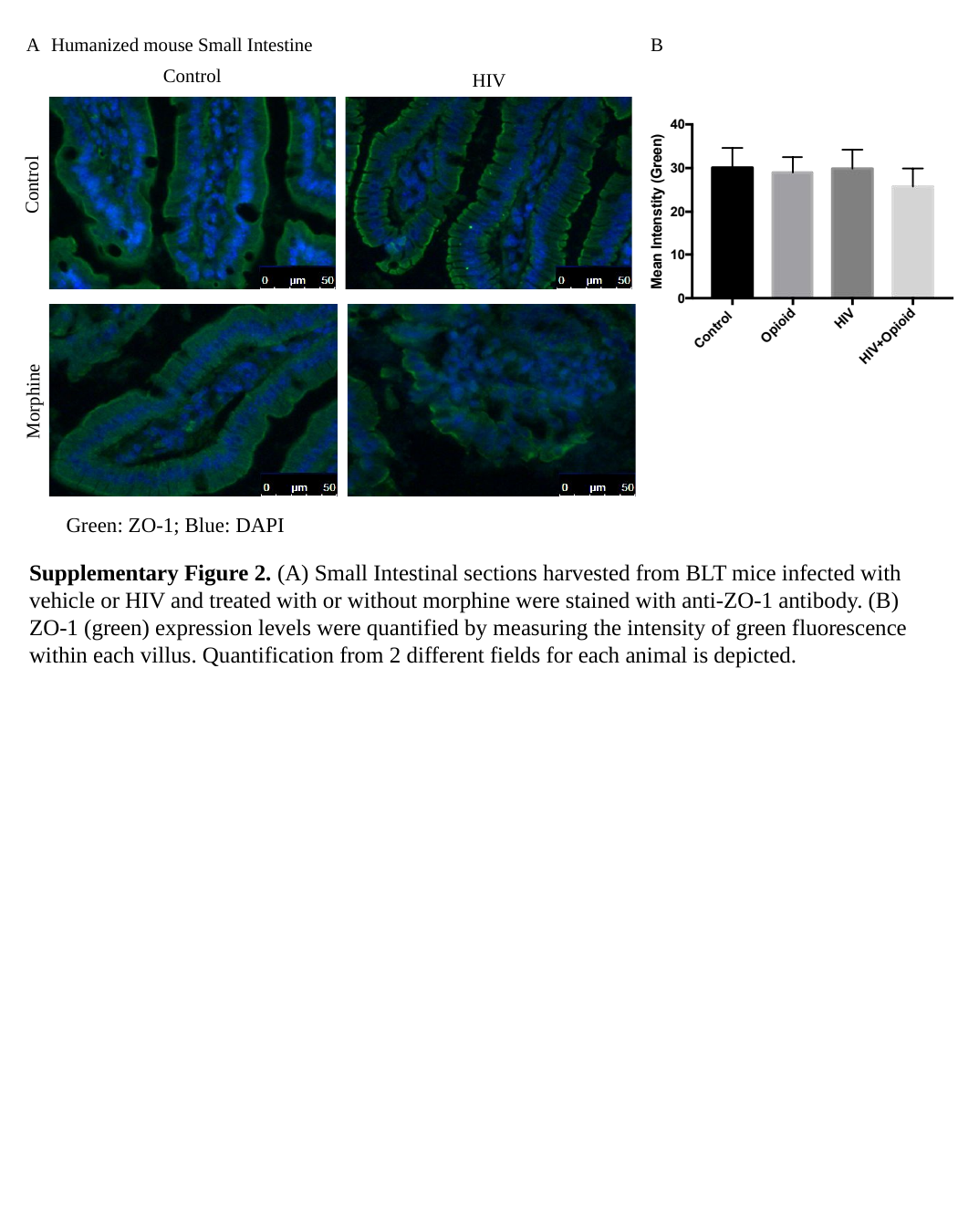

A
B
Humanized mouse Small Intestine
Control
HIV
Control
Morphine
Green: ZO-1; Blue: DAPI
Supplementary Figure 2. (A) Small Intestinal sections harvested from BLT mice infected with vehicle or HIV and treated with or without morphine were stained with anti-ZO-1 antibody. (B) ZO-1 (green) expression levels were quantified by measuring the intensity of green fluorescence within each villus. Quantification from 2 different fields for each animal is depicted.
