# Supplementary material for: Opioids Impair Intestinal Epithelial Repair in HIV-Infected Humanized Mice
Source: Front Immunol. 2020 Jan 17;10:2999. doi: 10.3389/fimmu.2019.02999 (PMC6978907; doi:10.3389/fimmu.2019.02999)
Supplement: Supplementary file 7 [file Presentation_3.PPTX]

## Slide 1
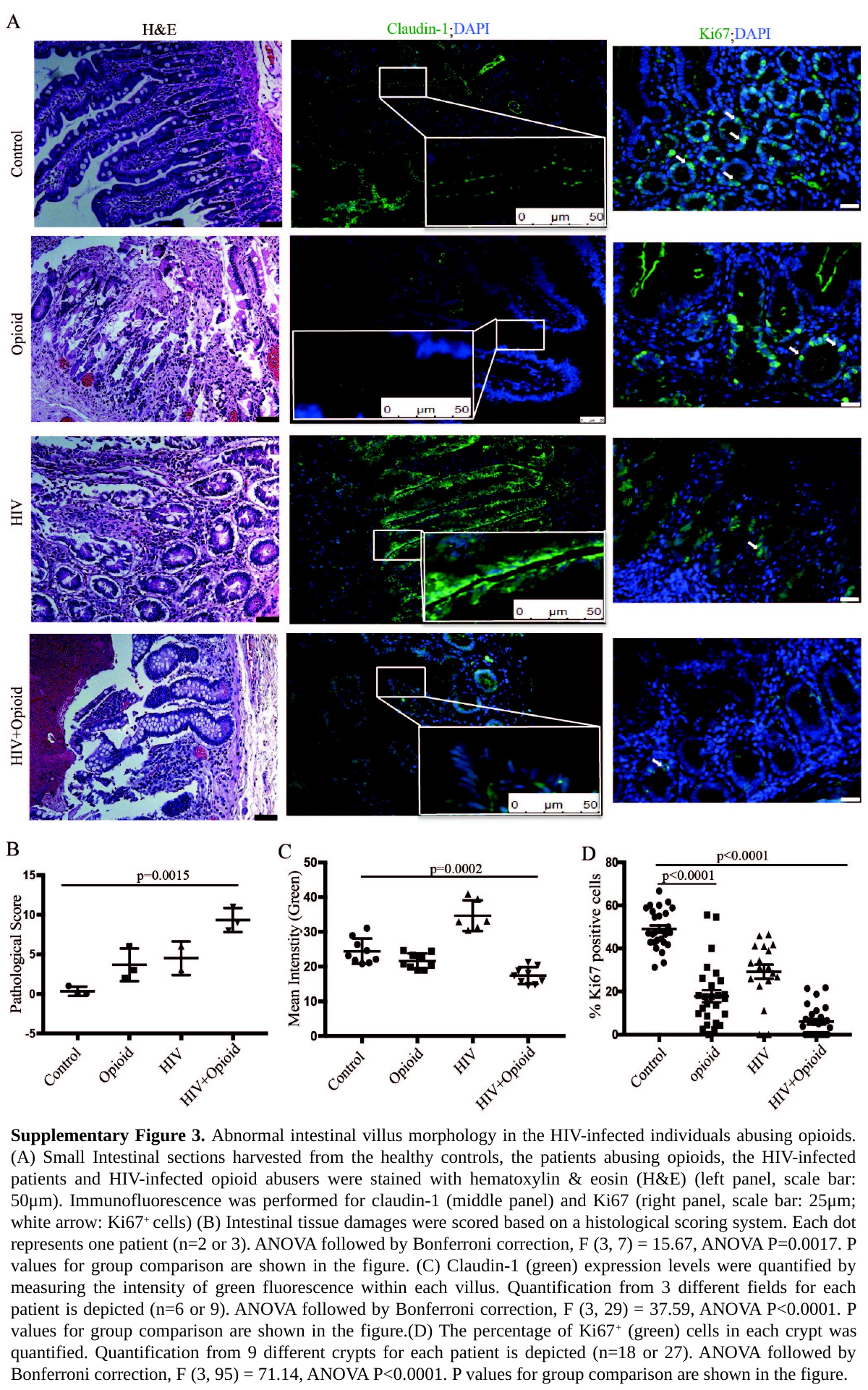

Supplementary Figure 3. Abnormal intestinal villus morphology in the HIV-infected individuals abusing opioids. (A) Small Intestinal sections harvested from the healthy controls, the patients abusing opioids, the HIV-infected patients and HIV-infected opioid abusers were stained with hematoxylin & eosin (H&E) (left panel, scale bar: 50μm). Immunofluorescence was performed for claudin-1 (middle panel) and Ki67 (right panel, scale bar: 25μm; white arrow: Ki67+ cells) (B) Intestinal tissue damages were scored based on a histological scoring system. Each dot represents one patient (n=2 or 3). ANOVA followed by Bonferroni correction, F (3, 7) = 15.67, ANOVA P=0.0017. P values for group comparison are shown in the figure. (C) Claudin-1 (green) expression levels were quantified by measuring the intensity of green fluorescence within each villus. Quantification from 3 different fields for each patient is depicted (n=6 or 9). ANOVA followed by Bonferroni correction, F (3, 29) = 37.59, ANOVA P<0.0001. P values for group comparison are shown in the figure.(D) The percentage of Ki67+ (green) cells in each crypt was quantified. Quantification from 9 different crypts for each patient is depicted (n=18 or 27). ANOVA followed by Bonferroni correction, F (3, 95) = 71.14, ANOVA P<0.0001. P values for group comparison are shown in the figure.
