# Supplementary material for: Opioids Impair Intestinal Epithelial Repair in HIV-Infected Humanized Mice
Source: Front Immunol. 2020 Jan 17;10:2999. doi: 10.3389/fimmu.2019.02999 (PMC6978907; doi:10.3389/fimmu.2019.02999)
Supplement: Supplementary file 8 [file Presentation_4.PPTX]

## Slide 1
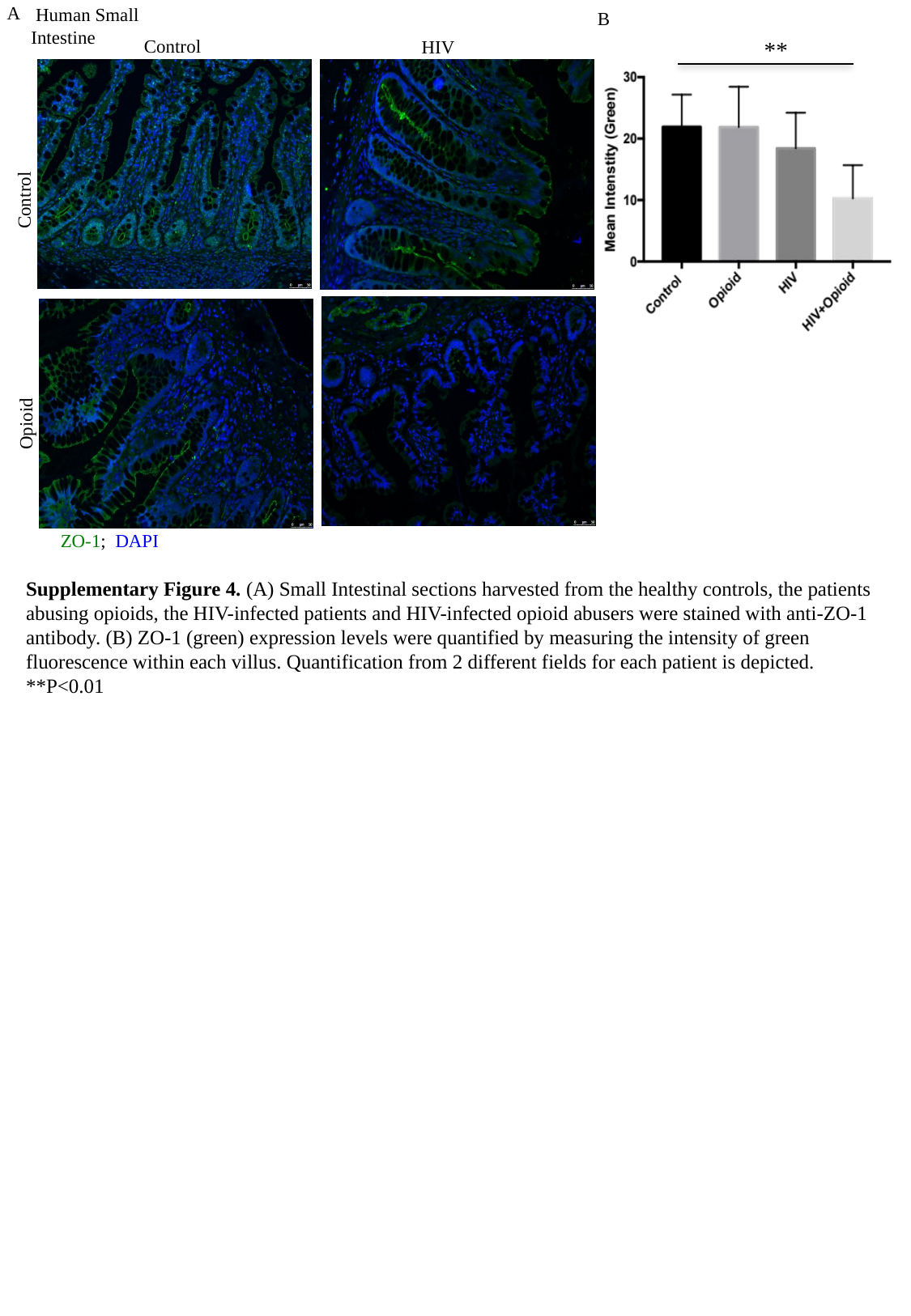

A
 Human Small Intestine
B
Control
**
HIV
Control
Opioid
ZO-1; DAPI
Supplementary Figure 4. (A) Small Intestinal sections harvested from the healthy controls, the patients abusing opioids, the HIV-infected patients and HIV-infected opioid abusers were stained with anti-ZO-1 antibody. (B) ZO-1 (green) expression levels were quantified by measuring the intensity of green fluorescence within each villus. Quantification from 2 different fields for each patient is depicted. **P<0.01
