# Supplementary material for: Opioids Impair Intestinal Epithelial Repair in HIV-Infected Humanized Mice
Source: Front Immunol. 2020 Jan 17;10:2999. doi: 10.3389/fimmu.2019.02999 (PMC6978907; doi:10.3389/fimmu.2019.02999)
Supplement: Supplementary file 9 [file Presentation_5.PPTX]

## Slide 1
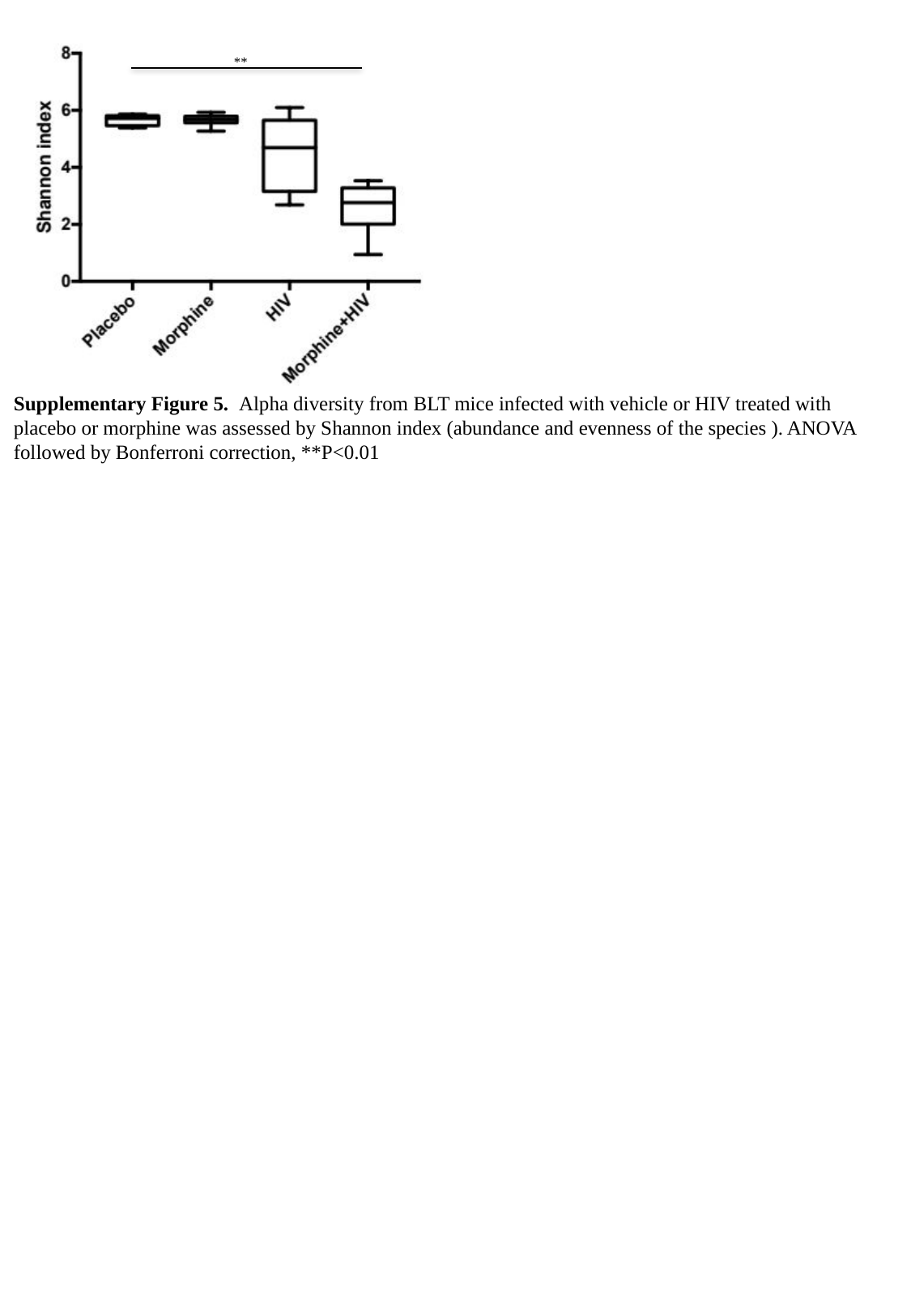

**
Supplementary Figure 5. Alpha diversity from BLT mice infected with vehicle or HIV treated with placebo or morphine was assessed by Shannon index (abundance and evenness of the species ). ANOVA followed by Bonferroni correction, **P<0.01
