# Supplementary material for: Opioids Impair Intestinal Epithelial Repair in HIV-Infected Humanized Mice
Source: Front Immunol. 2020 Jan 17;10:2999. doi: 10.3389/fimmu.2019.02999 (PMC6978907; doi:10.3389/fimmu.2019.02999)
Supplement: Supplementary file 10 [file Presentation_6.PPTX]

## Slide 1
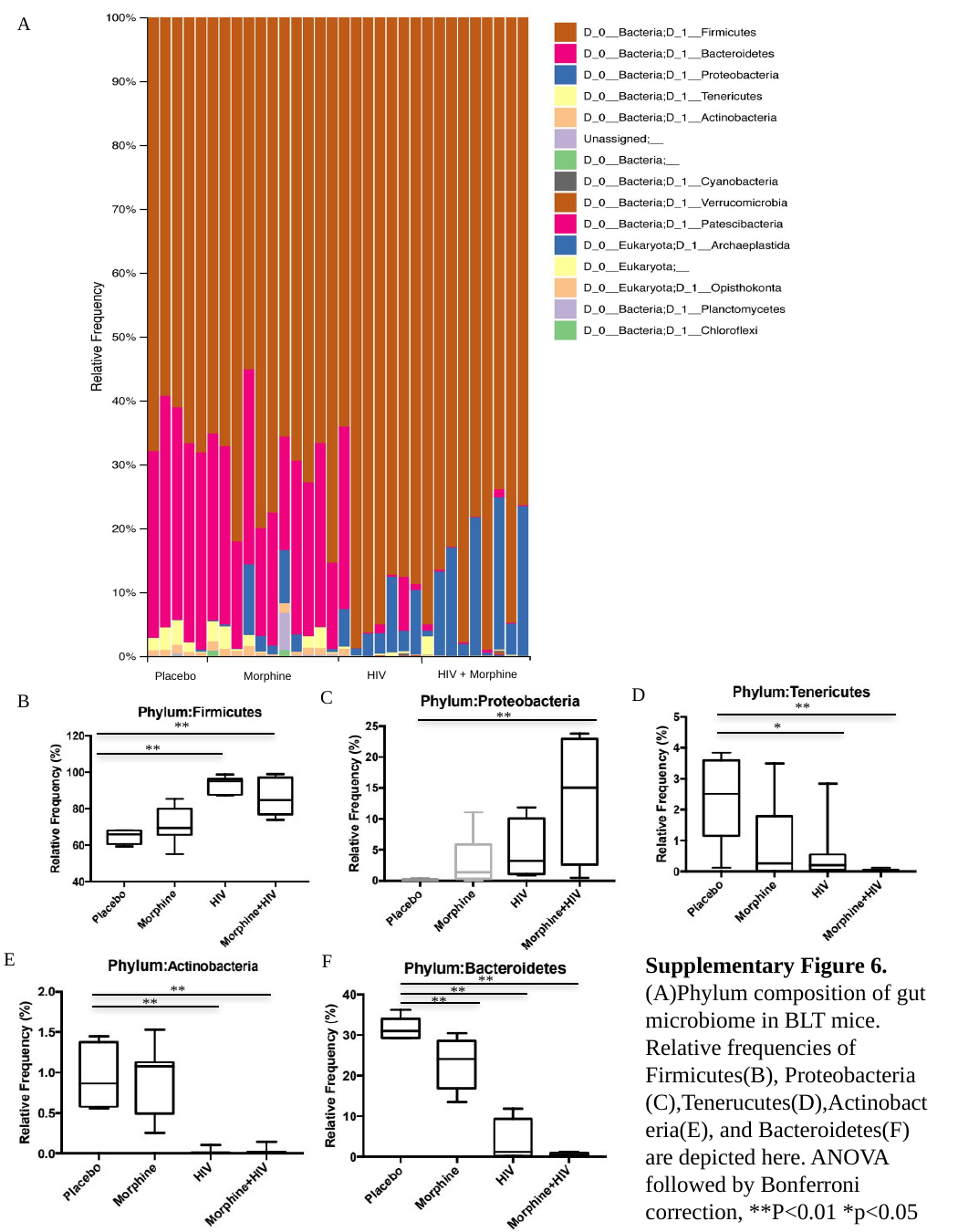

A
HIV + Morphine
 HIV
Morphine
Placebo
D
C
B
**
**
**
 *
**
E
F
Supplementary Figure 6. (A)Phylum composition of gut microbiome in BLT mice. Relative frequencies of Firmicutes(B), Proteobacteria (C),Tenerucutes(D),Actinobacteria(E), and Bacteroidetes(F) are depicted here. ANOVA followed by Bonferroni correction, **P<0.01 *p<0.05
**
**
**
**
**
