# Supplementary material for: Opioids Impair Intestinal Epithelial Repair in HIV-Infected Humanized Mice
Source: Front Immunol. 2020 Jan 17;10:2999. doi: 10.3389/fimmu.2019.02999 (PMC6978907; doi:10.3389/fimmu.2019.02999)
Supplement: Supplementary file 11 [file Presentation_7.PPTX]

## Slide 1
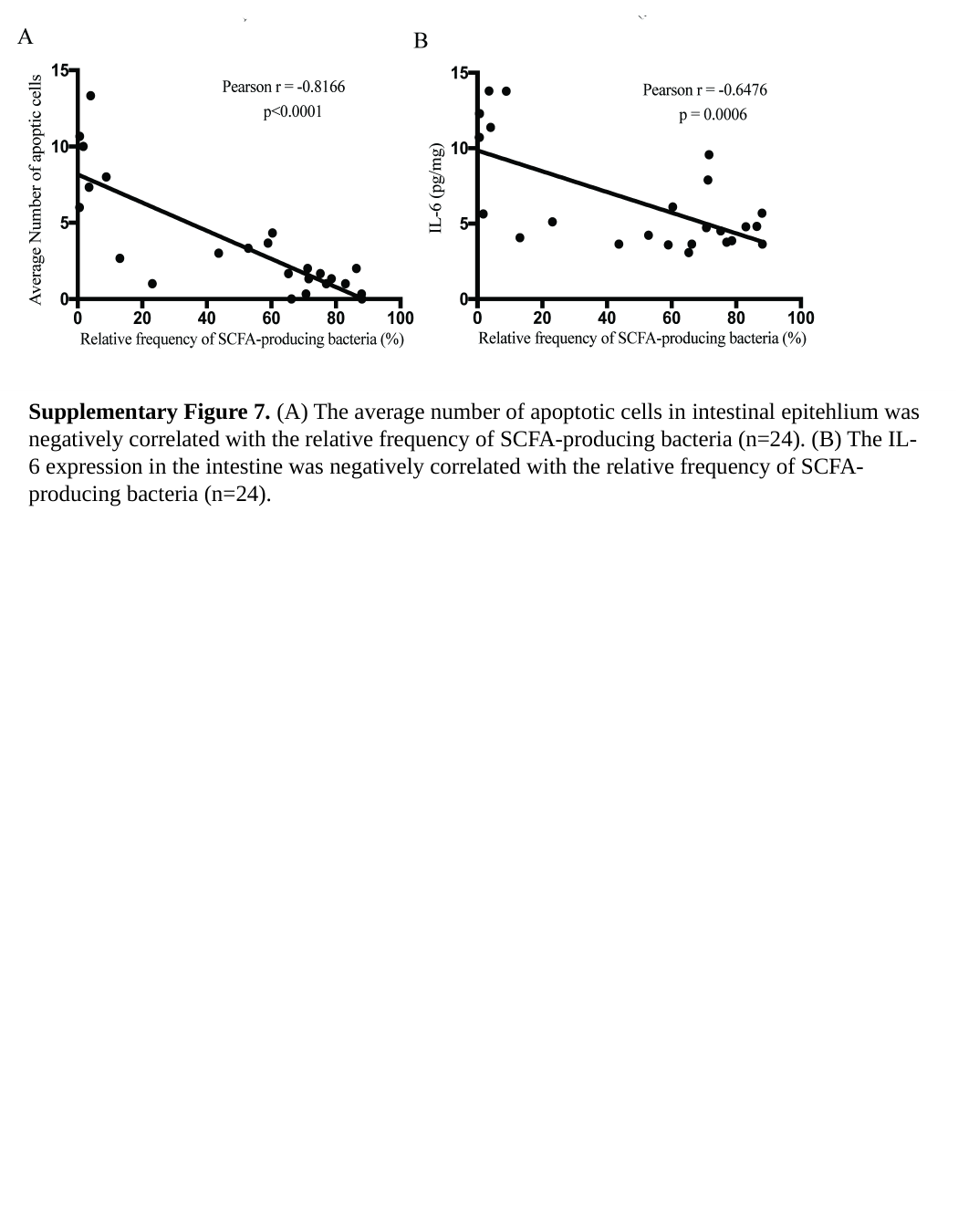

Supplementary Figure 7. (A) The average number of apoptotic cells in intestinal epitehlium was negatively correlated with the relative frequency of SCFA-producing bacteria (n=24). (B) The IL-6 expression in the intestine was negatively correlated with the relative frequency of SCFA-producing bacteria (n=24).
