# Supplementary material for: Opioids Impair Intestinal Epithelial Repair in HIV-Infected Humanized Mice
Source: Front Immunol. 2020 Jan 17;10:2999. doi: 10.3389/fimmu.2019.02999 (PMC6978907; doi:10.3389/fimmu.2019.02999)
Supplement: Supplementary file 12 [file Presentation_8.PPTX]

## Slide 1
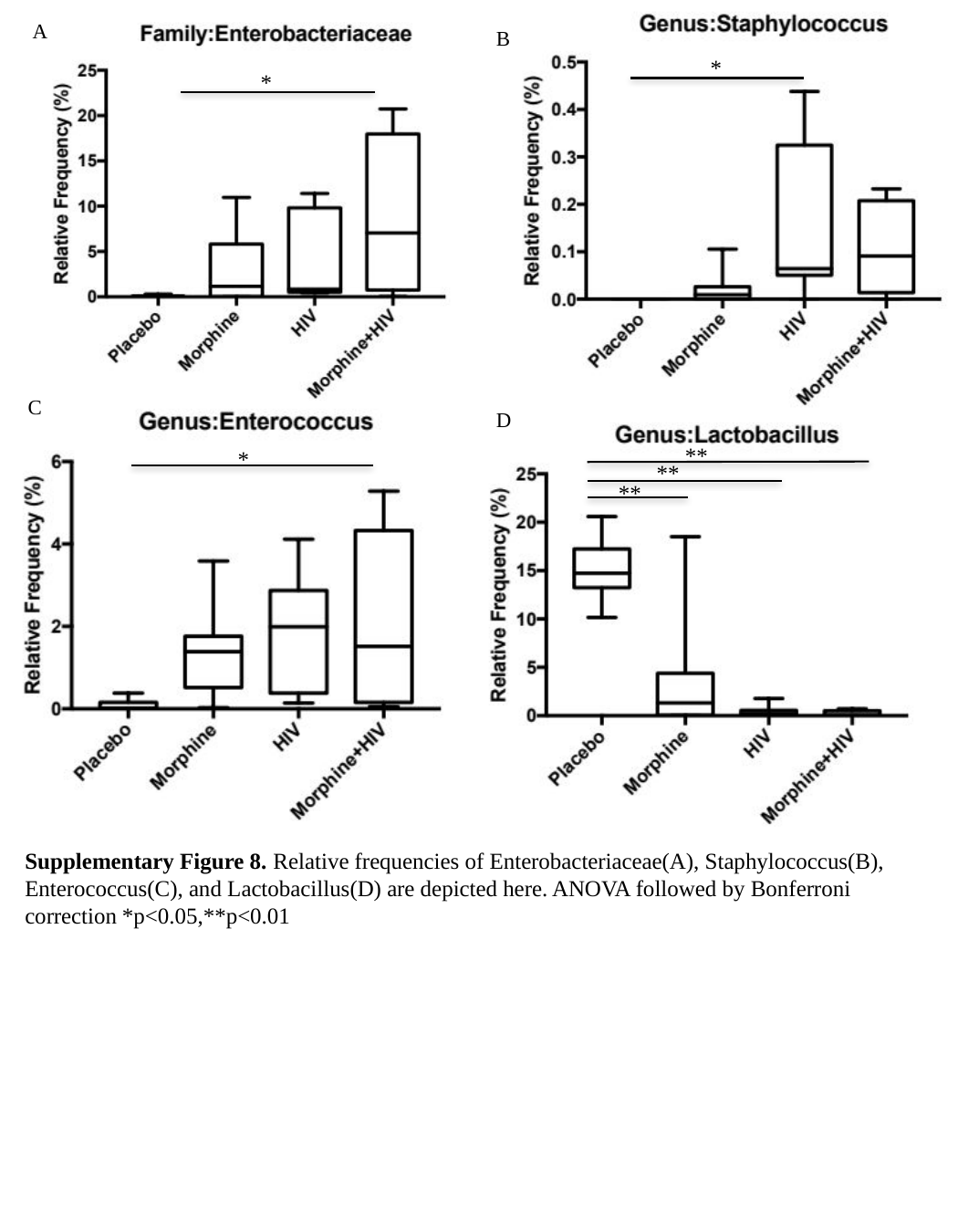

A
B
*
*
C
D
**
*
**
**
Supplementary Figure 8. Relative frequencies of Enterobacteriaceae(A), Staphylococcus(B), Enterococcus(C), and Lactobacillus(D) are depicted here. ANOVA followed by Bonferroni correction *p<0.05,**p<0.01
