# Supplementary material for: Opioids Impair Intestinal Epithelial Repair in HIV-Infected Humanized Mice
Source: Front Immunol. 2020 Jan 17;10:2999. doi: 10.3389/fimmu.2019.02999 (PMC6978907; doi:10.3389/fimmu.2019.02999)
Supplement: Supplementary file 13 [file Presentation_9.pptx]

## Slide 1
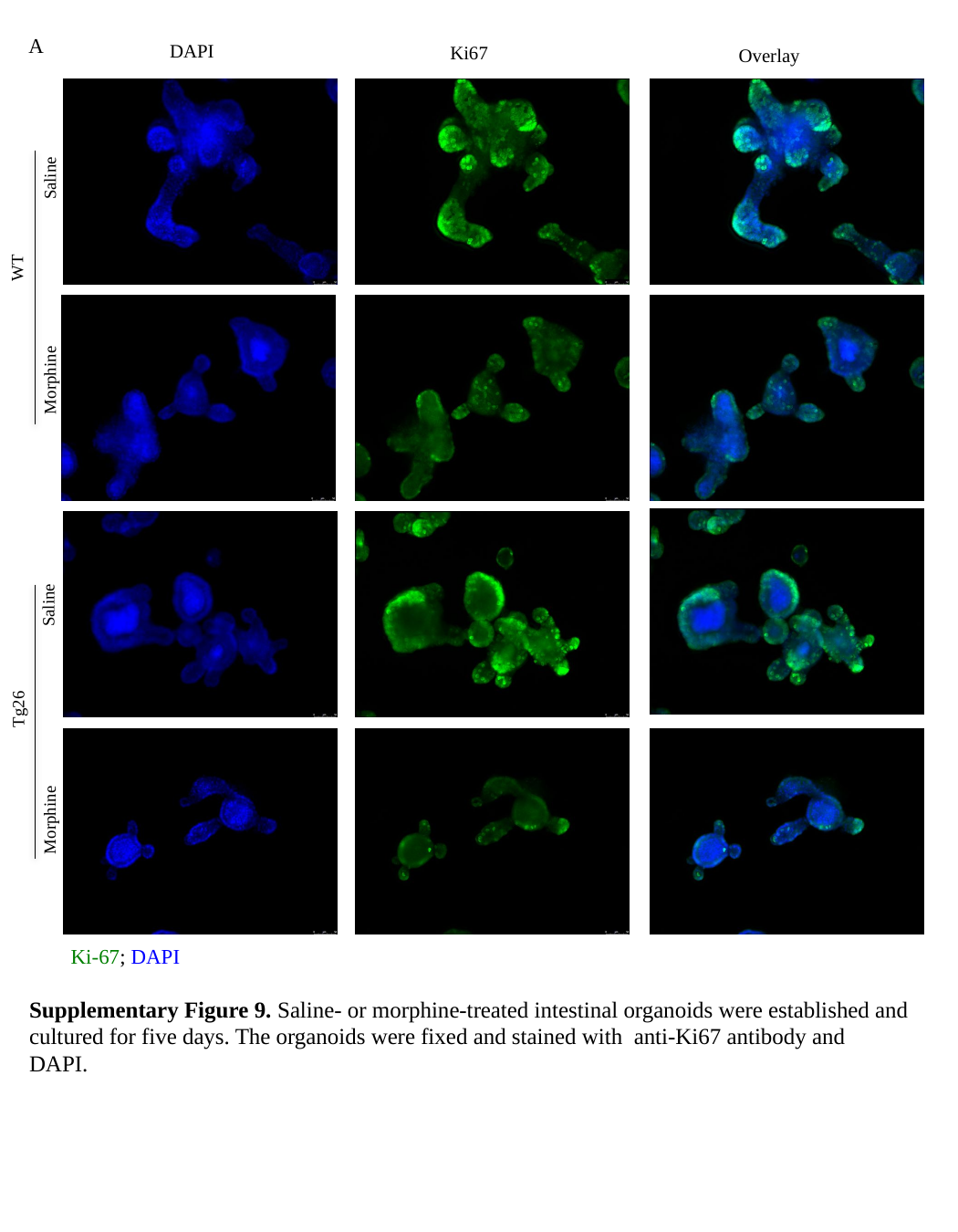

A
DAPI
Ki67
Overlay
Saline
WT
Morphine
Saline
Tg26
Morphine
 Ki-67; DAPI
Supplementary Figure 9. Saline- or morphine-treated intestinal organoids were established and cultured for five days. The organoids were fixed and stained with anti-Ki67 antibody and DAPI.
