# Supplementary material for: Opioids Impair Intestinal Epithelial Repair in HIV-Infected Humanized Mice
Source: Front Immunol. 2020 Jan 17;10:2999. doi: 10.3389/fimmu.2019.02999 (PMC6978907; doi:10.3389/fimmu.2019.02999)
Supplement: Supplementary file 14 [file Presentation_10.PPTX]

## Slide 1
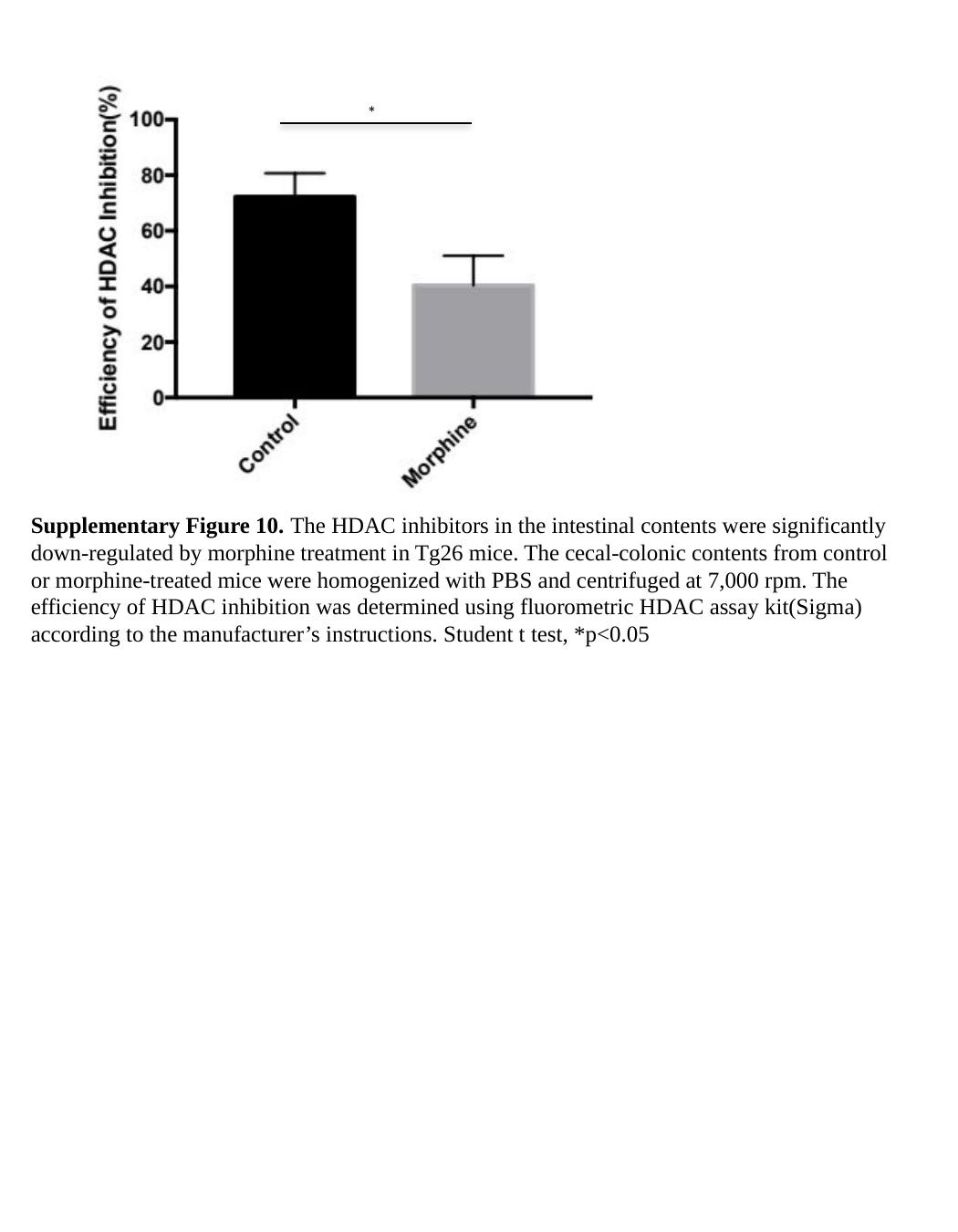

*
Supplementary Figure 10. The HDAC inhibitors in the intestinal contents were significantly down-regulated by morphine treatment in Tg26 mice. The cecal-colonic contents from control or morphine-treated mice were homogenized with PBS and centrifuged at 7,000 rpm. The efficiency of HDAC inhibition was determined using fluorometric HDAC assay kit(Sigma) according to the manufacturer’s instructions. Student t test, *p<0.05
