# Supplementary material for: Opioids Impair Intestinal Epithelial Repair in HIV-Infected Humanized Mice
Source: Front Immunol. 2020 Jan 17;10:2999. doi: 10.3389/fimmu.2019.02999 (PMC6978907; doi:10.3389/fimmu.2019.02999)
Supplement: Supplementary file 15 [file Presentation_11.PPTX]

## Slide 1
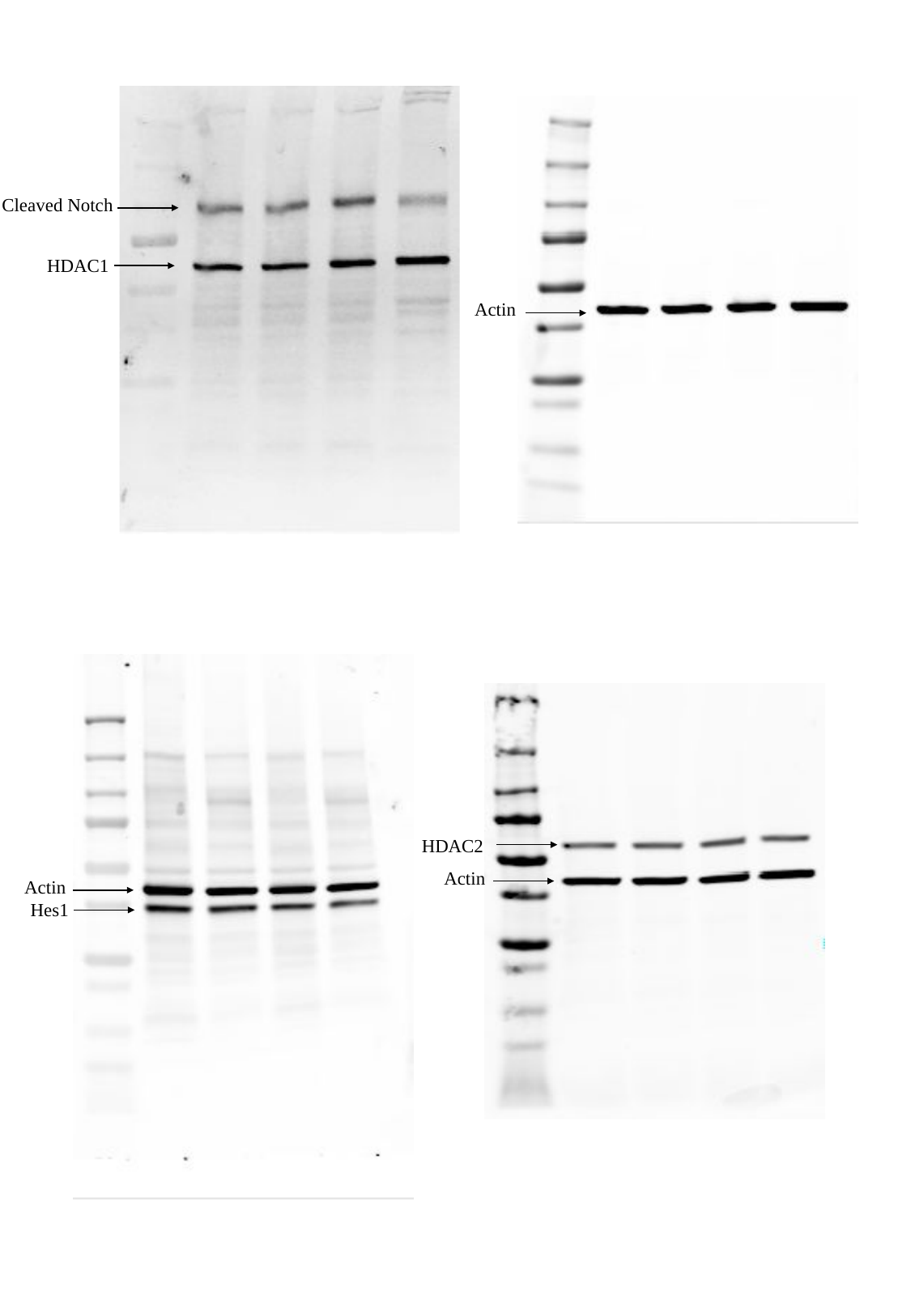

Cleaved Notch
HDAC1
Actin
HDAC2
Actin
Actin
Hes1

## Slide 2
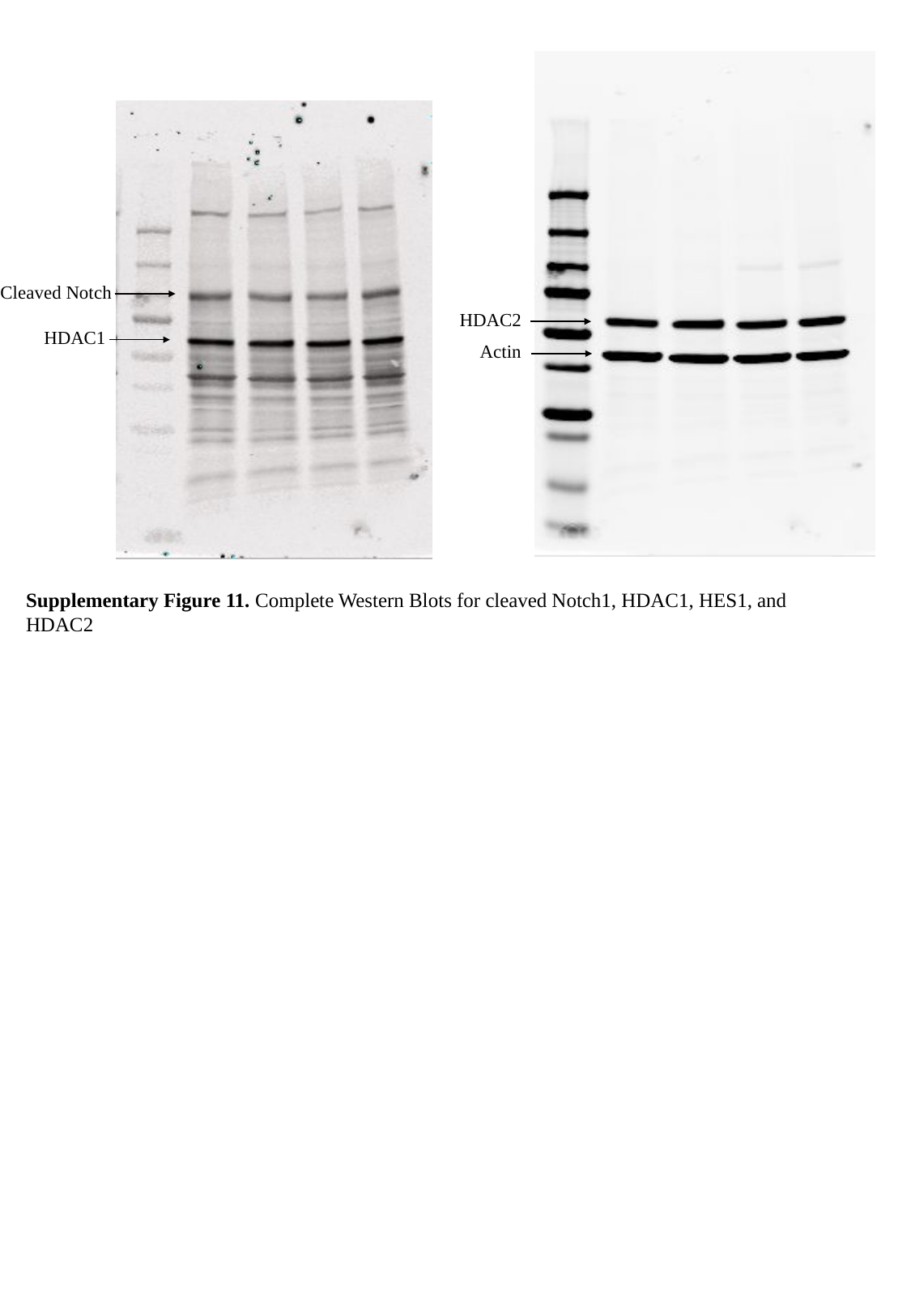

Cleaved Notch
HDAC2
HDAC1
Actin
Supplementary Figure 11. Complete Western Blots for cleaved Notch1, HDAC1, HES1, and HDAC2
