# Supplementary material for: Opioids Impair Intestinal Epithelial Repair in HIV-Infected Humanized Mice
Source: Front Immunol. 2020 Jan 17;10:2999. doi: 10.3389/fimmu.2019.02999 (PMC6978907; doi:10.3389/fimmu.2019.02999)
Supplement: Supplementary file 16 [file Data_Sheet_1.PDF]

Supplementary Table1 Patient information

	Age	Sex	HIV	Drug use
Opioids_1	26	Male	Negative	IVDU-Cocaine and heroin
Opioids_2	33	Female	Negative	Cocaine and Heroin abuse
Opioids_3	27	Male	Negative	Vicodin&percocet; Cocaine; MOLLY; acid; marijuana; mushrooms
HIV_1	66	Male	Positive	Cannabinoids
HIV_2	59	Male	Positive	None
HIV+Opioids_1	39	Male	Positive	Past IVDA heroin
HIV+Opioids_2	25	Male	Positive	IVDA reported
HIV+Opioids_3	60	Male	Positive	IVDA, benzoes and meth, administered morphine at hospital
